# Glass Frit Jetting for Advanced Wafer-Level Hermetic Packaging

**DOI:** 10.3390/ma15082786

**Published:** 2022-04-11

**Authors:** Ali Roshanghias, Jochen Bardong, Alfred Binder

**Affiliations:** Silicon Austria Labs GmbH, Europastrasse 12, A-9524 Villach, Austria; jochen.bardong@silicon-austria.com (J.B.); alfred.binder@silicon-austria.com (A.B.)

**Keywords:** wafer-level jetting, hermetic packaging, MEMS packaging, dispensing, glass frit bonding

## Abstract

Glass frit bonding is a widely used technology to cap and seal micro-electromechanical systems on the wafer level using a low melting point glass. Screen printing is the main method to apply glass frit paste on wafers. Screen printing of glass frit paste is usually performed on less sensitive, less critical wafers, normally the capping wafer, because screen printing is a rough process involving the mechanical contact of the screen printing mesh and the wafer. However, for some applications in which contactless patterning of glass frit materials on the device wafers are preferred (e.g., 3D topographies, micro-lens and optics integration) jet dispensing could be a promising approach. Consequently, in this study, wafer-level jetting of glass frit materials on silicon wafers was proposed and investigated. The jetting parameters such as jetting distance, power and temperature were optimized for a glass frit paste. Additionally, the effect of jetted pitch size on the bond-line thickness was assessed. The wafers with jetted glass frit pastes were conclusively bonded in low vacuum and characterized. As a single-step (non-contact) additive approach, the jet printing of glass frit was revealed to be a straightforward, cost-effective and flexible approach with several implications for hermetic packaging.

## 1. Introduction

Hermetic packaging via wafer bonding is a preferred approach for micro-electromechanical systems (MEMS), since it decreases the packaging complexity during production, reduces the fabrication costs and enhances the lifetime of the components. Wafer bonding is widely used in MEMS to create cavities at the wafer level, while glass frit wafer bonding is a standard technology for the hermetical encapsulation of MEMS [1,2]. It has been used to manufacture accelerometers, pressure sensors, micro-pumps, tactile sensors and flow sensors [3]. The glass frit pastes usually consist of finely ground glass frits with a particle size < 15 µm and an organic binder. Glass frit bonding has several advantages over other capping technologies such as hermeticity, less sensitivity to surface roughness and topographies, compatibility to metallic lead-throughs, usability with most materials, high process yield and low fabrication costs. On the contrary, glass frit bonds also had shortcomings in terms of the wide bonding rim (>200 µm) and the possible introduction of contaminations (Pb, Bi, etc.) to the components [4,5,6,7,8,9].

Glass frit bonding is performed in three steps: (a) the deposition of the glass frit paste via screen printing, (b) pre-treatment of the paste (burn-out of the organic binders and pre-glazing) and (c) finally bonding under pressure. Screen printing is the standard method for applying glass frit pastes. Screen printing of glass frit paste is normally carried out on the less sensitive wafer, i.e., the capping wafer; because screen printing is a rough process involving the mechanical contact of the screen printing mesh with the wafer. During screen printing, the glass frit is spread in front of the squeegee and then applied to the cap wafer via the squeegee to push the glass frit through the screen. The main advantage is the possibility of material deposition on cap wafers without additional effort, like photolithography. The possible feature sizes for screen-printed glass frit paste are in the range of <150–200> µm with a minimum spacing of 100 µm. The printed height of the screen-printed glass paste is about 30 µm [10,11]. For instance, in a study, 2D micro-mirrors have been successfully sealed under vacuum by means of glass frit bonding methods. A bond frame of a minimum of 300 µm was necessary in order to achieve a stable vacuum of 200–2000 Pa in the package without getter even more than 5 months after dicing [12].

Nonetheless, for some applications, glass frit materials cannot be applied on the cap wafers, for instance, when the cap wafer cannot stand glass frit process steps before bonding, or due to possible contamination with the organics or temperature shocks. Therefore, glass frit should be applied on the MEMS device, which is a bottleneck for screen printing due to possible damage to sensitive structures upon mechanical contact. In these scenarios, a contactless printing method is essential. Additionally, for some niche applications, in which contactless patterning of glass frit materials on the device wafers are preferred (e.g., 3D topographies, micro-lens and optics integration), contactless printing could be a promising approach [10,13,14,15]. Additionally, the pattern-distortion vulnerability and material-wasting of the expensive pastes make screen printing incapable of meeting the requirement of low-cost packaging. Regarding contactless printing, Liu et al. proposed an electrospraying process to deposit glass frit materials [10]. A uniform glass frit film with a line width within the range of 400–500 µm was prepared when the speed of the substrate was 25 mm/s. In our previous study, inkjet printing of adhesives was suggested as a promising contactless alternative to screen printing or needle dispensing for MEMS packaging [16]. Several UV-curable hybrid adhesives were examined, while the low viscosity of the adhesives and their jet-ability were considered as the most crucial parameters for inkjet printing. However, this viscosity range (<0.02 Pa.s) is too low for glass frit paste, so inkjet printing cannot be implemented here [17,18]. Alternatively, in this study, a contactless jet dispensing technology was employed which can handle viscose pastes. Different jetting parameters were studied and optimized for glass frit dispensing which will be discussed in the following chapters.

## 2. Materials and Methods

**Figure 1:**
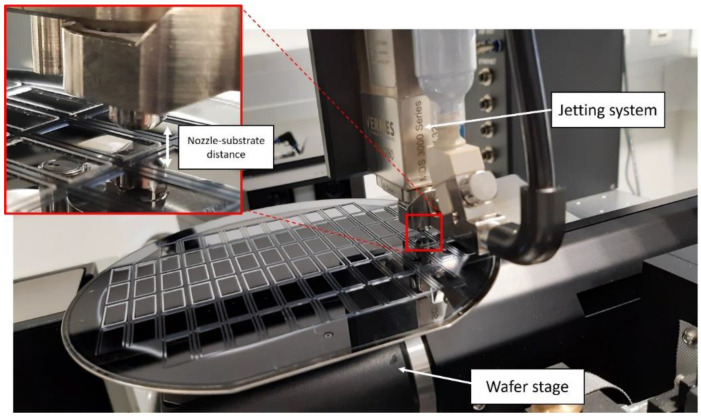
Two optical images of the wafer-level glass frit jetting in action.

A semi-automatic micro-assembly station (Häcker automation, Holzkirchen, Germany) was equipped with a jet dispensing system (MDS 3280, VERMES Microdispensing GmbH, Holzkirchen, Germany [19]) and used for glass frit jetting. The jetting valve was based on the piezoelectric actuator and equipped with a tappet with a conical tip. Figure 1 shows the experimental setup for the wafer-level jetting of glass frit paste at two magnifications. Here, the constructed stage for precise placement of wafers up to 200 mm can be seen. Additionally, the jetting system which contains both the control electronics and the piezo actuator is presented. The tappet inside the valve is actuated up and down, pushing the medium through the nozzle opening. The glass frit paste is located inside the pressurized container which supplies fluid through the chamber to the nozzle insert. The key advantage of this system compared to an inkjet printer is that it supports highly viscous materials with a viscosity of up to 2000 Pa.s. The deposition of droplets is triggered via the serial interface to the micro-assembly station’s software. Some jetting parameters can be manipulated directly and some indirectly [19].

Experiments were designed to find the right process parameters for glass frit jetting. Three nozzles with different opening sizes (i.e., 50 µm, 70 µm and 100 µm) were tested at different jetting impulse forces, while the nozzles could be heated from room temperature up to 80 °C. The effects of jetting distance, jetting power, temperature and nozzle opening size on the minimum droplet size were investigated. For droplet analysis, single points were programmed and expanded to a 20-by-20 dot matrix with a 500 µm distance via the software’s substrate control interface. For each parameter, three samples were fabricated and analyzed. The glass paste used for this study was DL11-210 (Ferro Crop., Mayfield Heights, OH, USA [20]) with a particle size distribution of d10: 1–3 µm, d50: 6–8 µm and d90: 12–20 µm. The paste had a viscosity of 35 ± 15 Pa.s and a solid content of 87 ± 2%.

For the wafer-level jetting experiments, silicon cap wafers with a diameter of 150 mm and thickness of 500 µm were used. Here, cavities with a depth of 200 µm and area of L: 14.6 mm and W: 6.2 mm were dry-etched. Each cavity possessed a bond frame with a width of 500 µm. The glass frit paste was jetted on the bond frame at 4 different pitch sizes (center to center distance) of 200, 225, 250 and 300 µm with an average width of 225 µm. The printed structures were pre-treated in a convection oven at 430 °C. Afterward, glass wafers (MEMpax ^®^, Schott AG, Mainz, Germany)with a thickness of 200 µm were bonded to the cap wafers under a low vacuum (10 Pa) using a wafer bonder (EVG 520 IS, EV Group, Florian am Inn, Austria) at 460 °C. The bonding time and force were defined to be 30 min and 2000 N, respectively. The glass wafer had a coefficient of thermal expansion of 3.26 × 10^−6^/K. Raman spectroscopy was carried out to obtain qualitative information about the nitrogen content inside the cavities in comparison to the environment to determine the hermeticity of cavities [21,22]. A mechanical stylus profilometer (Dektak XT-A, Brucker, MA, USA) was also utilized to investigate the topography of the printed droplets and frames as well as the sealed cavities.

## 3. Results

### 3.1. Nozzle Distance Effect

**Figure 2:**
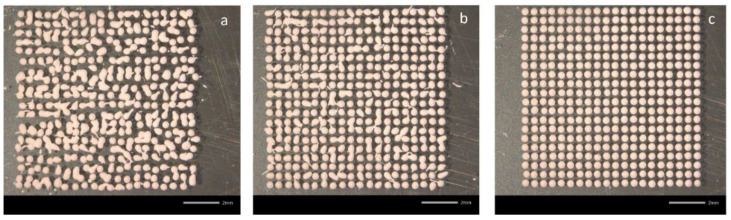
The effects of the nozzle to substrate distance: (**a**) 3 mm, (**b**) 2 mm and (**c**) 1 mm.

The jetting nozzle distance, which is the distance between the bottom edge of the nozzle and the substrate, was varied from 1 to 3 mm as shown in Figure 2. As inferred from this figure, by decreasing the distance, the jetting becomes more consistent, so do the droplet shapes and the distribution The reduction in splash and the increase in droplet position accuracy at 1 mm nozzle–substrate distance is also apparent in Figure 2. A further decrease in distance to 0.5 mm did not lead to any discernible improvement. Therefore, 1 mm was determined as the optimal nozzle distance.

### 3.2. Droplet Size Analysis

**Figure 3:**
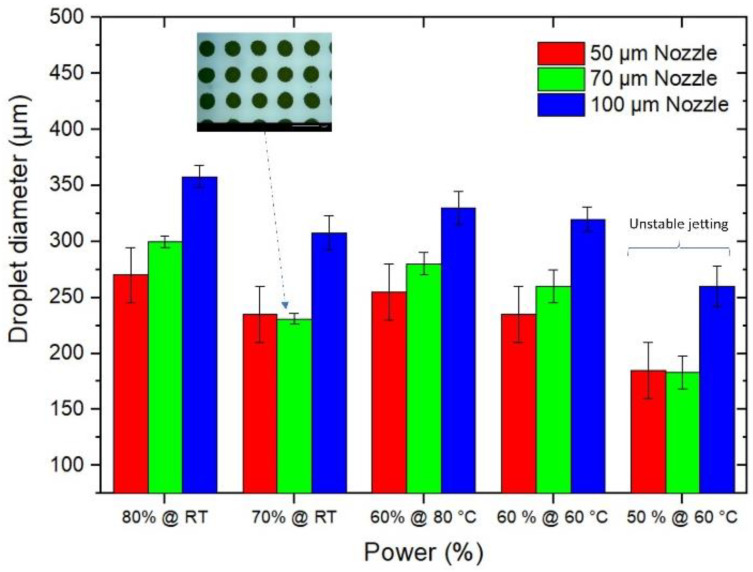
Droplet size analysis of the glass frit paste jetted with different nozzles, powers and temperatures.

Since the droplet size plays a big role in determining the resolution of the printed layer, the effects of jetting parameters and nozzle sizes on the average droplet size were primarily studied. In Figure 3 the effects of jetting power and nozzle temperature on the average droplet size are presented. The jetting valve power (in %) is the main input parameter of the controlling unit and can be set between 0% and 100%. This power corresponded to the impulse force with which the material is driven through the nozzle. The jetting of a viscous medium such as glass frit paste required relatively higher powers. The glass frit jetting at higher power ranges (≥70%) succeeded at room temperature, whereas for lower power ranges, a consistent jetting was only conceivable at elevated temperatures (i.e., 60 or 80 °C). Here, the higher temperature assisted the weaker impulse force by reducing the viscosity of the paste [17,23]. By looking again at Figure 3, however, one can see that increasing the temperature could result in bigger droplets.

It can be observed that by decreasing the jetting power, relatively smaller droplets were attained. On the other hand, lower powers required elevated temperatures, which shortened the shelf time of the paste and increased the risk of nozzle clogging. As a matter of fact, although the jetting of glass frit paste succeeded at a power span of 50 to 80%, the consistency and repeatability of printing trials were proven to be higher at 70% and 80% power. Additionally, Figure 3 shows that by decreasing the nozzle diameter, the average droplet size decreases; however, the differences between 100 µm and 70 µm nozzles were remarkedly higher than those between 70 µm and 50 µm. Since the change in nozzle clogging in a 50 µm nozzle was higher than 70 µm, the optimal nozzle size was defined to be 70 µm. Moreover, the 70% jetting power was selected above other jetting forces for glass frit paste, rendering an average droplet size of 225 ± 25 µm.

### 3.3. Pitch Size Analysis

**Figure 4:**
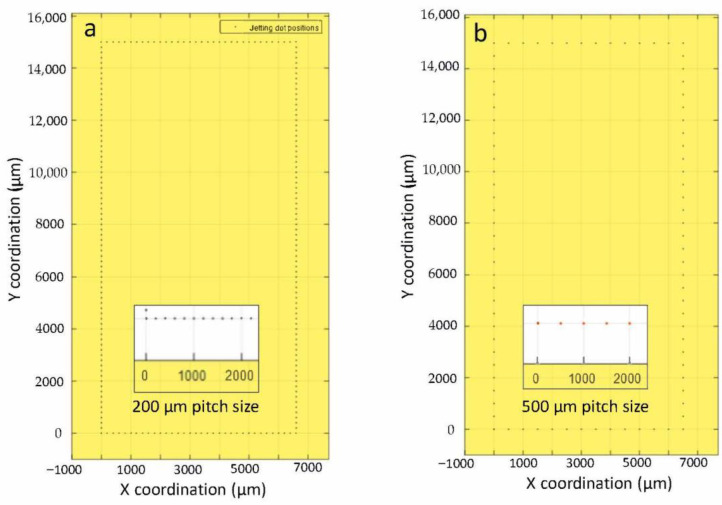
Two examples of the CAD files for jetting of the glass frit as a bond frame in two resolutions, i.e., 200 µm pitch size (**a**) and 500 µm pitch size (**b**). Here the pitch size is the center-to-center distance between two droplets.

**Figure 5:**
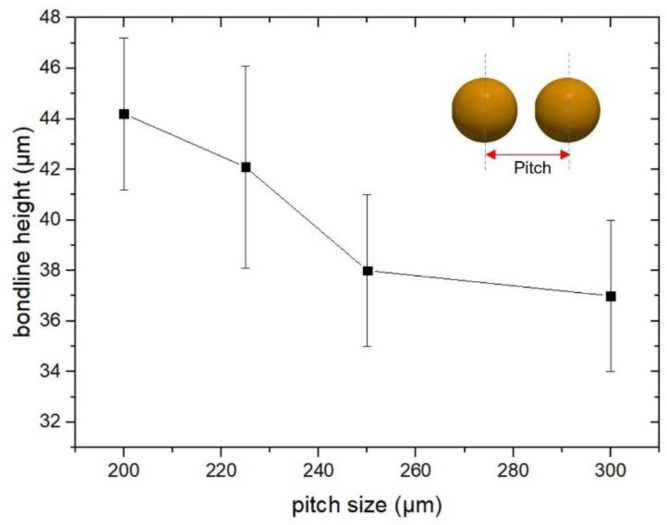
Bond frame height as a function of jetting pitch size.

**Figure 6:**
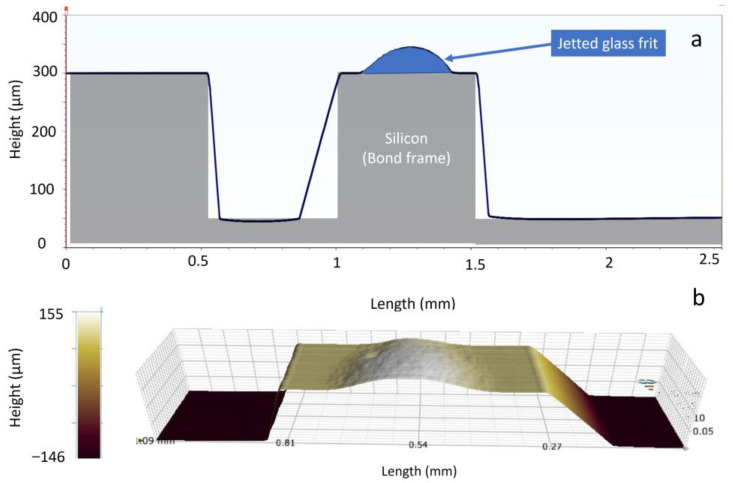
2D (**a**) and 3D (**b**) profilometric graphs of the cap silicon wafer with the jetted glass frit paste.

**Figure 7:**
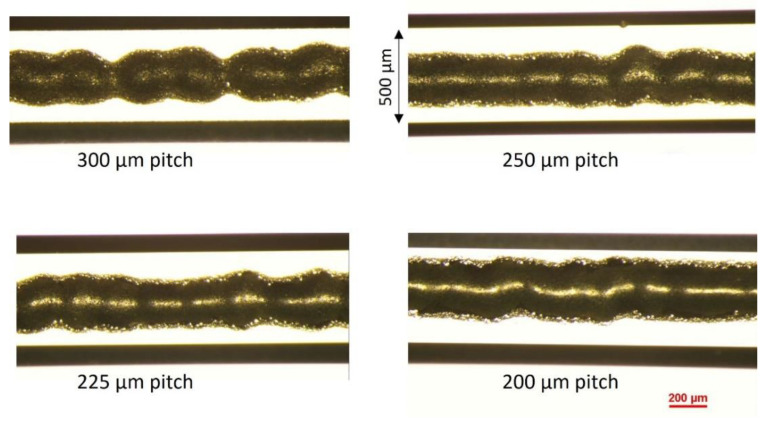
The effects of jetting pitch on the topography of the printed bond frame.

For generating a continuous bond frame, which guarantees the sealing of the resulting package, it is critical to define the required number of droplets. Assuming a droplet size of 225 ± 25 µm, it was planned to investigate different pitch sizes (center to center distance of two neighboring droplets) from 200 to 300 µm. In Figure 4, two examples of computer-aided design (CAD) for jetting the glass frit as a bond frame are depicted. Here the jetting was exercised on the actual cap wafers. Figs 5 and 6 present the profilometry results. One can comprehend from Figure 5 that by increasing the pitch size, the height of the resulting bond-line is reduced. The height of the jetted bond-line was 44 µm at a pitch size of 200 µm, whereas at a pitch size of 300, it was reduced to 37 µm. Here, we aimed to decrease the bond-line height while preventing large voids in the glass frit by missing material. Figure 6 shows two examples of 2D and 3D profilometry results. Additionally, in Figure 7, the printed bond frames at the different pitch sizes are exhibited. Figure 7 implies that the printed lines at a lower pitch size are indeed more uniform. It can be seen that the width of the printed lines also increased at lower pitch sizes.

In another study by Chen et al. [14], a nominal-actual comparison of the width of screen-printed glass frit materials by using a similar glass frit material (DL11-036) was pursued. In that study, by varying the screen opening dimensions from 100 to 400 µm, an actual width of from 147.8 to 515.6 µm was obtained. This means that the actual width of the glass frit was almost 30 to 47% higher than the nominal values. The height of the screen-printed pattern was in the range of 27 to 32 µm, which was decreased by half upon glazing and further reduced to 8–12 µm under the bonding pressure. All in all, it can be deduced that the jet-printed paste was in the same height/width range as the screen-printed ones. Moreover, Figure 7 indicates that continuous lines can be generated by jet printing.

**Figure 8:**
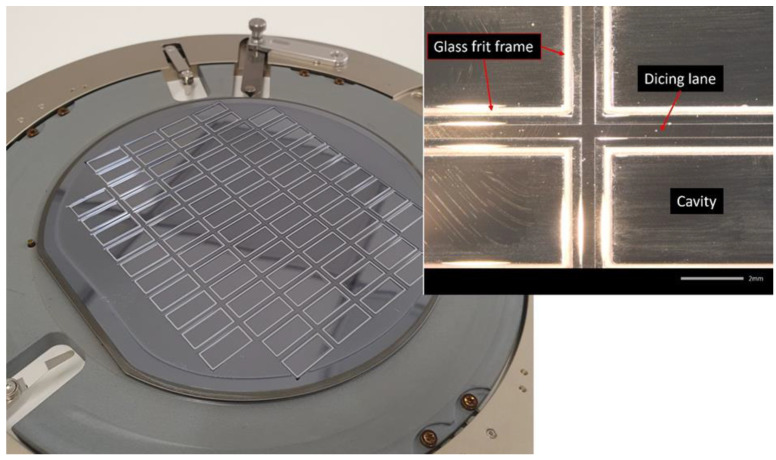
A demonstration of the 6″ silicon cap wafer with the jetted glass frit paste at different magnifications.

**Figure 9:**
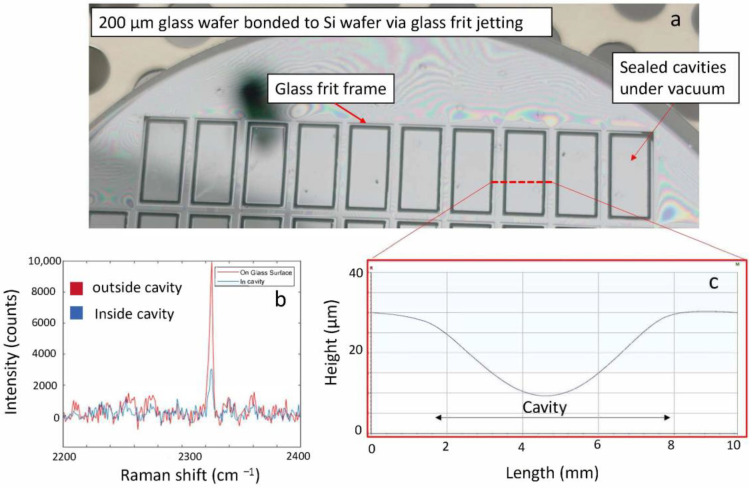
Glass frit wafer bonding results of the cap wafer bonded to a 200 µm thick glass wafer (**a**) optical image of the wafer implying the sealed cavities under low vacuum (10 Pa). (**b**) the Raman spectroscopy result indicating the hermeticity of the cavities in terms of N2 level. (**c**) shows the deflection of the thin glass wafer in the cavity area due to the pressure difference manifesting the hermeticity of the cavities.

Figure 8 shows the final configuration of the glass frit printed cap wafer. Here the glass-frit-jetted bond-line (rim), cavity and the dicing lanes are shown. In the final version, a MEMS device will be located inside of the glass frit sealing rim which is not shown here. The results of wafer bonding using the printed cap wafer are presented in Figure 9. As seen, the frames are bonded and the cavities are sealed, while the areas outside the cavities are not bonded. The Raman spectroscopy result in Figure 9b indicates the hermeticity of the cavities in terms of nitrogen content level. The profilometry analysis of the cavities also revealed the deflection of thin glass over the cavities, manifesting the pressure difference to ambient. All the bonded wafers rendered 100% yield without any detectable failure or voids.

## 4. Future Implications

In this study, the jet printing of a standard glass frit paste, made for screen printing, was successfully performed. The prospect of jetting a screen-printable glass frit paste spurs the development of dedicated jettable glass frit pastes. Recently, jettable solder pastes and surface mount pastes and adhesives have emerged in the market [24,25]. Similarly, modified glass frit paste for jet-dispensing should be introduced to gain further benefits. In fact, smaller droplet size, bond frame width and thickness can be anticipated with a customized jettable paste, which in turn can open up new possibilities and applications for glass frit bonding. Here, several advantages of jet printing of glass frit could be already comprehended such as the freedom in design, width and thickness of the paste. This is an issue and bottleneck for current state-of-the-art screen printing. Here, jet-dispensing can be the game-changer, where the volume and shape of the printed material can be designed and selectively applied. Moreover, as the distance between the jetting nozzle and substrate can be kept above 0.5 mm, the mobility of MEMS structures capped by glass frit bonding can be retained; therefore, glass frit printing can even be directly applied to the device wafer. As another example, Bargiel et al. [13] integrated microlenses with fragile movable parts of a silicon microactuator by the (needle) dispensing of several droplets of glass frit paste. Here, screen printing could not be utilized. For such applications, glass frit jetting can be considered the perfect solution. Last but not least, owing to the high-speed jetting of the paste with a frequency >3000 Hz, wafer-level jet dispensing yields high throughput and low production time. It is worth mentioning that in the current study, the jetting of the whole 6-inch wafer took several minutes (up to 5 min) since the jetting was done using a customized R&D setup and a single nozzle. However, by optimizing control software and the interface to the jetting valve, integrating multi-nozzles as well as utilizing an automated wafer handling, positioning and alignment unit, the jetting time could be reduced to a few seconds.

## 5. Conclusions

In this study, the jet dispensing of glass frit paste has been introduced and explored for wafer-level packaging. Although the frit material was formulated for screen printing, consistent jetting was successfully conducted at room temperature with an average droplet size of 225 µm. The optimum nozzle-to-wafer distance was revealed to be 1 mm, while the 70 µm nozzle with 70% jetting power rendered stable jetting with a small droplet size. The bond-line experiments indicated that by increasing the jetting pitch size, the height of the as-printed line decreased to 37 µm. The wafer bonding results verified the hermeticity of the sealed cavities. Unlike screen printing, the presented approach does not involve direct contact between the printing tool and the wafers. Additionally, the width and thickness of the bond frame can be engineered selectively and the printing can be done at high speed and high throughput. Several implications and further improvements have been suggested and discussed.

## Data Availability

Data are available upon request from authors.
